# A Case of Fatal Bleeding Following the Removal of Adenoid Vegetations

**Published:** 1898-02

**Authors:** 


### A CASE OF FATAL BLEEDING FOLLOWING THE REMOVAL OF ADENOID VEGETATIONS.

Dr. E. Schmiegelow reports in *Monatssch. f. Ohrenheilk.*, Jahrg. XXXI., No 3, the following very rare complication of the operation for the removal of adenoids of the naso-pharynx.

Primary bleeding, from the removal of adenoid vegetations, fortunately occurs rarely. Seldom are cases reported in literature where serious bleeding after this operation is met with (Bryson, Delavan, Cartaz, Woakes, Rualat, Gelle and Beasoleil). More commonly the bleeding has ceased before the occurrence of complete collapse.

Death in one case reported by Delavan, after a digital examination of the posterior nares, was without doubt due to the fact that the child was a "bleeder." The case reported by Schmiegelow was that of a boy 12 years old, treated at the polyclinic of Frederick's Hospital, Copenhagen. The patient complained of disability to breathe through his nose, and had to breathe through his mouth altogether. He was small for his age, always held his mouth open, while his nose appeared compressed laterally. On both sides of his neck, both before and behind the sterno-mastoid, there were swollen lymphatics. The family history of the patient was scrofulous. By digital examination it was ascertained that the vault of the naso-pharynx and posterior nasal meatuses were filled with adenoid vegetations. The exploring finger was somewhat bloody. The operation was performed with the patient seated, while an assistant steadied the head with one hand and with the other held the hands of the patient. The boy sat quietly during the performance of the operation, without struggling or without apparent fear. A Gottstein's curette was introduced into the naso-pharynx, a mouth-gag being used to hold open the mouth. First, by a stroke of the instrument it was carried into the middle line. The handle was passed to the left, so that the blade was carried into the right side of the naso-pharynx, when three or four sweeps were made. Immediately there ensued a very profuse hemorrhage from the mouth and nose, of bright arterial blood, without any preliminary dripping. It was seen at once that unless assistance were quickly rendered, danger of a serious nature was imminent. The patient meanwhile sinking from his seat, was assisted to a table, and a tampon of iodoform gauze introduced anteriorly and posteriorly. His breathing became rapid; he was very pale and cyanotic.

Bleeding was controlled after tampon was put in place, but in spite of subcutaneous and intravenous injections respiration was not re-established. An autopsy was performed, which resulted as follows: The heart and its adjacent veins were well filled with blood. (The patient had lain with his head lowered and the extremities had been wrapped with bandages.) All the internal organs were markedly anemic. The right lateral wall of the naso-pharynx was lacerated and there were clots of blood in the wound. There was found in the internal carotid a long wound, just below the portion which enters the carotid canal of the petrous portion of the bone. Besides this, there were few lesions of the vessels at the side of the wound of the pharyngeal wall. Numerous enlarged glands existed contiguous to the artery. The course of the vessel was microscopically normal.

An explanation of the manner in which the accident occurred was not apparent. It seemed certain that the serious hemorrhage was from a lesion of the internal carotid artery, and that the cause for the pharyngeal bleeding was the wound of the side-wall of the pharynx, through which the blood found an outlet. But how the internal carotid was ruptured is not clear. Possibly the great swelling of the glands to the connective tissue of the side of the neck had an influence upon the fatal result. The internal carotid was evidently injured through the pharyngeal wall.

The Gottstein's curette had in all probability pressed upon the lateral wall of the pharynx and brought so much force to bear upon the internal carotid that this artery was pressed tightly against the cranium, resulting in its rupture.—*American Medical Surgical Bulletin.*

